# Enhanced CO_2_ Hydrogenation to Methanol Using out‐of‐Plane Grown MoS_2_ Flakes on Amorphous Carbon Scaffold

**DOI:** 10.1002/smll.202408592

**Published:** 2025-02-10

**Authors:** Mo Lin, Maxim Trubyanov, Han Wei Lee, Artemii S. Ivanov, Xin Zhou, Pengxiang Zhang, Yixin Zhang, Qian Wang, Gladys Shi Xuan Tan, Kostya S. Novoselov, Daria V. Andreeva

**Affiliations:** ^1^ Institute for Functional Intelligent Materials Materials Science and Engineering Department National University of Singapore 4 Science Drive 2 Singapore 117544 Singapore

**Keywords:** amorphous carbon, chemical vapor deposition, CO_2_ hydrogenation, graphene oxide, MoS_2_

## Abstract

The conversion of excess carbon dioxide (CO_2_) into valuable chemicals is critical for achieving a sustainable society. Among various catalysts, molybdenum disulfide (MoS_2_) has demonstrated potential for CO_2_ hydrogenation to methanol. However, its catalytic activity has yet to be fully optimized, and scalable, industrially viable production methods remain underdeveloped. In this work, a chemical vapor deposition (CVD) approach is introduced to grow vertically oriented MoS_2_ crystals on an amorphous carbon template. This method enhances the exposure of vacancy‐rich basal planes, which are crucial for stable catalytic performance. The 2H‐MoS_2_ flakes, supported on a conductive carbon scaffold, exhibit catalytic activity, achieving a net space‐time yield of 2.68 g_MeOH_ g_cat_⁻¹ h⁻¹ with a selectivity of 82.5% under mild conditions (264 °C, 10 bar). This work highlights a significant step toward the industrial application of MoS_2_‐based catalysts for CO_2_ conversion, bridging the gap between fundamental research and scalable implementation.

## Graphical abstract

A novel catalytic platform for carbon dioxide (CO_2_) hydrogenation to methanol is developed using vertically grown, sulfur‐vacancy‐rich molybdenum disulfide (MoS_2_) on amorphous carbon scaffolds. This method achieves enhanced catalytic efficiency with high selectivity under mild conditions. The approach offers a sustainable pathway for CO_2_ conversion and highlights the potential of tailored 2D materials in industrial catalysis applications.

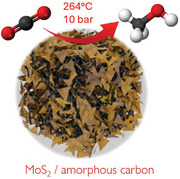

## 1 Introduction

The climate warming and increasing demand for sustainable energy sources have intensified research efforts to develop efficient methods for converting carbon dioxide (CO_2_) into value‐added fuels.^[^ ^1^, ^2^, ^3^, ^4^ ^]^ The catalytic hydrogenation of CO_2_ to methanol is a component of carbon capture and utilization strategies, which aim to recycle CO_2_ emissions from industrial processes into valuable products, rather than releasing it into the atmosphere.^[^ ^5^, ^6^ ^]^ Traditionally, methanol is produced from fossil fuels like natural gas. Hydrogenation of CO_2_ offers a more sustainable pathway to produce methanol, reducing reliance on non‐renewable resources.

Methanol can serve as a convenient liquid carrier for hydrogen, which is otherwise difficult to store and transport. This process converts hydrogen and CO_2_ into a stable, transportable liquid, which can be re‐converted to hydrogen when needed. The creation of an energy‐efficient catalytic process necessitates the development of high‐performance catalysts that can facilitate CO_2_ hydrogenation at low temperatures, thereby conserving energy.^[^ ^7^ ^]^ However, despite the numerous metal oxide catalysts proposed, many continue to face challenges such as short life cycles and diminished activity at lower temperatures. For instance, catalysts like In_2_O_3_ and ZnO/ZrO_2_, along with some newly developed metal oxides, often require temperatures exceeding 300 °C to achieve optimal catalytic activity for CO_2_ hydrogenation, resulting in increased energy consumption.^[^ ^8^, ^9^, ^10^ ^]^ While the introduction of catalysts based on Pd,^[^ ^11^, ^12^, ^13^, ^14^ ^]^ La^[^ ^15^ ^]^ and Ce^[^ ^16^, ^17^ ^]^ has shown promise, there are concerns regarding the long‐term stability and increased expense.

Significant progress has been made in catalyzing the hydrogenation reaction of CO_2_ into methanol using sulfur vacancy‐rich molybdenum disulfide (MoS_2_).^[^ ^7^ ^]^ Sulfur vacancies in both the basal plane and adges are crucial for enhancing the adsorption and activation of CO_2_ molecules, thereby facilitating subsequent chemical transformations. While exposed edges are well‐known for boosting catalytic activity in other applications, such as the hydrogen evolution reaction,^[^ ^18^, ^19^ ^]^ their stability in CO_2_ hydrogenation presents challenges. Specifically, the edges promote over‐hydrogenation, leading to the formation of undesired methane, which reduces both catalytic efficiency and selectivity.^[^ ^7^ ^]^ This highlights the importance of selectively activating the basal planes while minimizing the influence of edge sites to enhance performance in CO_2_ hydrogenation to methanol, improve stability, and optimize catalytic efficiency in related processes.

In this study, we developed a template‐assisted chemical vapor deposition (CVD) method to synthesize supported nanostructured MoS_2_ with largely exposed, vacancy‐rich basal planes. Traditional approaches to MoS_2_ vertical growth aim to maximize the exposure of edge sites^[^ ^20^, ^21^ ^]^ only, typically using silicon substrates.^[^ ^22^ ^]^ This vertical stacking occurs due to the higher diffusion rate of sulfur through van der Waals gaps compared to across the basal planes.^[^ ^23^ ^]^ By tuning CVD growth parameters, the preferential formation of vertical MoS_2_ structures over horizontal layers was achieved.^[^ ^24^ ^]^ In this work, we advanced this process by using an amorphous carbon support, which increases nucleation site density,^[^ ^25^ ^]^ thereby regulating the orientation of MoS_2_ layers.

The porous amorphous carbon template was created through the pyrolysis of a resin‐graphene oxide (GO) core–shell network, providing a high surface area of 394 m^2^ g^−1^, which supports of chaotic nucleaction and out‐of‐plane growth of 2H‐MoS_2_ crystals. We further evaluated the catalytic performance of this catalyst in CO_2_ hydrogenation, achieving a space‐time yield of 2.68 g_MeOH_g_cat_^−1^ h^−1^ under 264 °C. This performance surpasses many existing CO_2_ hydrogenation catalysts and demonstrates high stability.

## 2 Result and Discussion

### 2.1 Synthesis of Porous Carbon Scaffold

**Figure 1:**
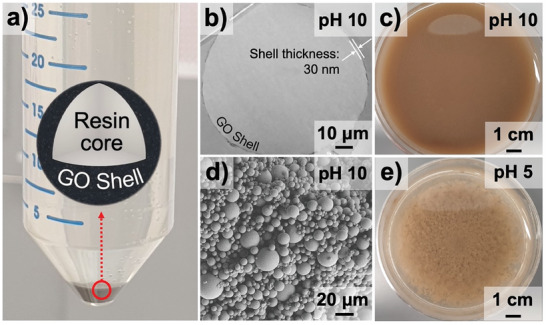
Preparation and characterization of GO‐resin microspheres. a) Digital photo of GO‐stabilized core‐shell microspheres obtained via resin‐in‐water emulsion templating with high‐intensity probe sonication after centrifugation. The insert illustrates the schematic of core/shell particles. b) Transmission electron microscopy (TEM) cross‐sectional images of GO‐resin core–shell microspheres prepared at pH 10. c). Digital photo of GO‐stabilized resin‐in‐water emulsions before centrifugation. Microdroplets form a homogeneous dispersion at pH 10. d) Scanning electron microscopy (SEM) image of individual microspheres prepared at pH 10. e) Digital photo illustrates the agglomeration of microspheres after pH adjustment to 5.

To fabricate GO‐resin core–shell particles, we mixed 250 mL of deionized water and 10 g of UV‐curable polyurethane/acrylate‐based resin. Initially, polyurethane/acrylate‐based resin is not miscible with water. To form an emulsion, we added 50 mL of aqueous GO dispersion (0.4 wt.%) and sonicated the mixture using a probe sonicator at 20 kHz and 0.33 W (power density of 0.5 W mL^−1^) for 6 min. The oxidized regions of the GO flakes form hydrophilic domains, while sp^2^ carbon regions form hydrophobic domains. Due to this hydrophilic/hydrophobic balance, GO flakes are self‐organized at the interfaces in the water/resin mixture, forming shells around the resin cores (**Figure** **1**a–c).

pH adjustment allows for control over the thickness of GO shells and interaction between microspheres. First, we adjusted pH to 10 by adding 0.1 m KOH using a potentiometric approach. At pH 10, the GO functional groups of the GO flakes are fully deprotonated, resulting in a negative charge on GO flakes. The electrostatic repulsion between the similarly charged GO flakes leads to the formation of 30‐nm‐thick GO shells (Figure 1b) and the stabilization of individual microspheres (Figure 1c). The prepared emulsion, containing individual microspheres, was centrifuged at 1000 rpm for 30 min to remove excess water and subsequently cured under UV light at 405 nm for 5 min while stirring. This process yielded a dispersion of core–shell microspheres with diameters ranging from 5 to 50 µm, consisting of resin cores and GO shells (Figure 1d).

**Figure 2:**
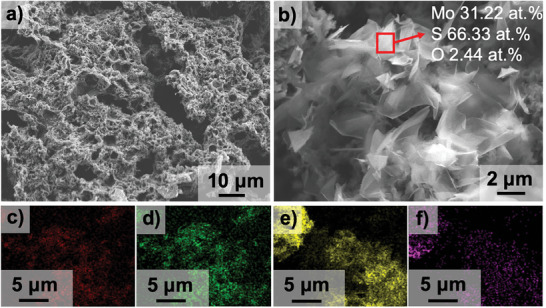
Out‐of‐plane growth of MoS_2_ flakes on carbon scaffold (CS). a) The SEM image of CS after pyrolysis. The insert shows a sketch of CS. b) The SEM image of MoS_2_ flakes on the surface of CS. The insert shows a schematic illustration of the MoS_2_/CS. c–f) The EDX mapping of S (red), Mo (green), C (yellow)and O (lila) in MoS_2_/CS.

Next, the pH of the dispersion was potentiometrically adjusted to 5 using 0.1 m HCl to initiate the agglomeration of GO‐coated microspheres into an interconnected network (Figure 1e). The agglomerates were then collected on a polyethersulfone membrane via vacuum‐assisted filtration. The dried at ambient conditions, interconnected microspheres were pyrolyzed in a tube furnace at 700 °C, with a heating rate of 3 °C min^−1^, for 2 h under an argon flow rate of 250 sccm. This pyrolysis process decomposed the resin cores and produced a 3D porous carbon scaffold (CS) (**Figure** **2**a).

During pyrolysis, the GO shell shrinks, forming a crumpled, interconnected network, as observed in the scanning electron microscopy (SEM) image (Figure 2a). It can be found in the SEM image of pores with sizes of several micrometers, which increase the surface area. This network exhibited a high Brunauer–Emmett–Telle (BET) surface area of 394 m^2^ g^−1^ (Figure S1, Supporting Information), indicating the removal of the resin core. The 4‐point probe method showed that this material possessed an high electrical conductivity of 50 ± 10 S m^−1^ (Figure S2, Supporting Information), higher than the value of activated carbon (41.8 S m^−1^),^[^ ^26^ ^]^ revealing that the GO shell was well reduced. Additionally, energy dispersive X‐ray (EDX) spectroscopy revealed that after thermal reduction, the CS contained less than 5 at.% oxygen (Figure S3, Supporting Information).

### 2.2 Growing Out‐of‐Plane mos_2_ Flakes on Amorphous Carbon Scaffold by CVD

The CVD method was employed to grow out‐of‐plane MoS_2_ flakes on the as‐prepared CS. The process utilized a two‐zone tubular furnace, with MoO_3_ and sulfur serving as the metal and chalcogen sources, respectively. Fine‐tuning the parameters — including the positioning of the MoO_3_ source relative to the CS, as well as the temperature and carrier gas flow rate — was crucial for the successful out‐of‐plane growth of MoS_2_ flakes. Specifically, the MoO_3_ and the CS were placed in the same boat, while the sulfur powder was positioned upstream in a separate boat. This setup ensured precise control over the deposition process, leading to the desired MoS_2_ morphology.

The optimal positioning for the MoO_3_ powder was determined to be 1 mm upstream of the CS within the same temperature zone, while the sulfur source was placed 45 cm upstream in another temperature zone. The argon purging flow rate was maintained at 90 sccm under atmospheric pressure. A heating rate of 20 °C min^−1^ was used for the MoO_3_ zone whose target temperature is 750 °C, while a slower rate of 3 °C min^−1^ was applied for the sulfur zone targeting at 150 °C. This temperature was held constant for 20 min after reaching the set value, followed by a cooling period. Upon completion of the CVD process, MoS_2_ flakes were observed to have grown out‐of‐plane on the CS. The final product, referred to as MoS_2_/CS, serves as the composite catalyst (Figure 2b).

To characterize the chemical composition of MoS_2_ flakes, we performed elemental analysis using energy dispersive X‐ray (EDX) spectroscopy (Figure 2b–f). The quantitative EDX results indicated that the oxygen concentration on the MoS_2_ flakes was less than 10 at.%, while the atomic concentrations of Mo and S were 31.22 at.% and 66.33 at.%, respectively. More quantitative EDX results were provided in Figure S4a (Supporting Information), and all these data reveal a low oxygen percentage and a 1:2 ratio between Mo and S. This ratio suggests a thorough reaction between sulfur and MoO_3_ during the CVD process, resulting in the successful formation of MoS_2_ crystals. In addition, the EDX mapping of S (Figure 2c) and Mo (Figure 2d) showed similar shapes while C (Figure 2e) and O (Figure 2f) showed similar shapes, further confirming that the complete conversion of molybdenum oxide and a slight amount of oxygen comes from the substrate.

Furthermore, to confirm the MoS_2_ loading on the CS, we conducted thermogravimetric analysis (TGA) measurement (Figure S5, Supporting Information) on both the CS and MoS_2_/CS. The weight losses observed were 16.1 wt.% for the CS and 22.9 wt.% for MoS_2_/CS, indicating that the MoS_2_ content in the MoS_2_/CS composite is 6.8 wt.%.

**Figure 3:**
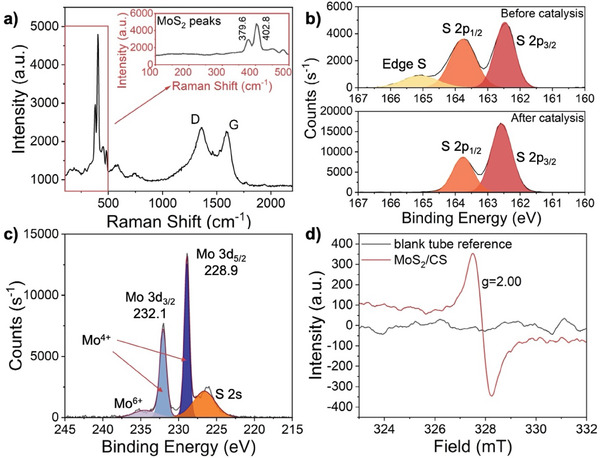
Raman, XPS and ESR characterization of MoS_2_/CS catalyst. a) The Raman spectrum of MoS_2_/CS catalyst. b) The XPS narrow scan of Mo 3d peak of MoS_2_/CS catalyst. c) The XPS narrow scan of S 2p peak of MoS_2_/CS catalyst before and after hydrogen (H_2_) reduction pretreatment and the catalytic process. d) ESR curves of blank reference and MoS_2_/CS catalyst.

In the Raman spectra (**Figure** **3**a), the broad carbon peak at 1350 cm^−1^ (D band) from CS indicates that the most of the carbon atoms are sp^3^ hybridized,^[^ ^27^ ^]^ confirming the amorphous nature of the scaffold. The MoS_2_ peaks are observed at 379.6 and 402.8 cm^−1^, corresponding to the E^2^g and A^1^g modes, respectively, which are characteristic of MoS_2_.^[^ ^28^ ^]^ According to the relationship between peak position and layer numbers of suspended MoS_2_,^[^ ^29^ ^]^ we can estimate that the MoS_2_ flakes are ≈3 layers thick.

The X‐ray photoelectron spectroscopy (XPS) analysis was performed on the MoS_2_ catalyst as well. The survey scans are put in the supporting material (Figure S6, Supporting Information). A narrow scan of Mo 3d peaks (Figure 3b) reveals that the majority of Mo atoms in our composite have a valency of +4, with only a small proportion of Mo atoms is in the +6‐valency state. The peak positions at 232.1 and 228.9 eV indicate that the MoS_2_ is in the 2H phase,^[^ ^30^ ^]^ which is the most suitable phase for the CO_2_ hydrogenation. The 1T phase, on the other hand, is less stable under high‐temperature conditions.^[^ ^31^ ^]^

The XPS narrow scan of the sulfur 2p peak (Figure 3c) shows peaks at 163.5 and 162.1 eV. A peak ≈165 eV indicates that the as‐grown MoS_2_ flakes have an excess of sulfur atoms,^[^ ^28^ ^]^ which is not favorable for the CO_2_ hydrogenation reaction, as sulfur vacancy plays a key role in the catalysis process.^[^ ^7^ ^]^ To address this, we performed hydrogen (H_2_) reduction pretreatment of the MoS_2_/CS before CO_2_ hydrogenation to create sulfur vacancies. The composite was exposed to a continuous flow of pure H_2_ gas, and the reduction process was conducted at an elevated temperature of 300 °C for 3 h. The quantitative XPS analysis revealed that after pretreatment the S/Mo ratio decreased from 2.32 before the pretreatment to 1.53 afterward.

Furthermore, electron spin resonance (ESR) analysis was carried out on the H_2_ pretreated MoS_2_. The g‐factor of ≈2 (g ≈ 2) observed in the ESR spectrum (Figure 3d) is attributed to sulfur vacancies, where the absence of a sulfur atom leaves behind an unpaired electron.^[^ ^32^ ^]^ These sulfur vacancies are crucial active sites for CO_2_ hydrogenation, playing a significant role in the catalytic process.

**Figure 4:**
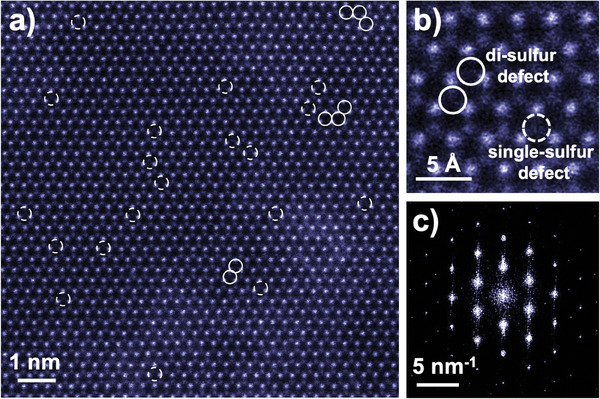
STEM/HAADF analysis of MoS_2_/CS catalyst. a) STEM/HAADF image of the monolayer 2H‐MoS_2_, with dashed and solid circles highlighting single sulfur and di‐sulfur defects, respectively. b) Enlarged STEM image cropped from a), clearly showing the di‐sulfur and single‐sulfur defects. c) Fourier transform pattern of the STEM image.

To have a better understanding of sulfur vacancies in MoS_2_/CS catalyst, scanning transmission electron microscope/high‐angle annular dark‐field (STEM/HAADF) analysis was carried out. **Figure** **4**a presents the STEM/HAADF image of MoS_2_/CS catalyst. In this figure, the bright dots are Mo atoms, and the dim dots are S atoms. Contrast reduction at sulfur sites can be found where sulfur vacancies are located. Not only single‐sulfur defects but also di‐sulfur defects were found in the STEM image. Figure 4b shows a clearer enlarged STEM image providing the difference of the two kind point defects. It has been reported that di‐sulfur vacancies can accelerate the reduction of CO_2_ due to its ability to decrease the energy barrier for the conversion of COOH^∗^ to CO^∗[^ ^33^, ^34^ ^]^ In addition, the Fourier transformation pattern was extracted from the STEM image (Figure 4c), confirming the hexagonal lattice structure (2H phase) of the material.

### 2.3 Hydrogenation of CO_2_ and Selectivity Toward Methanol Production

The catalytic performance of MoS_2_/CS for the hydrogenation of CO_2_ was evaluated in a packed bed tubular reactor. As previously mentioned, the catalyst was reduced in a flow of H_2_ gas at 300 °C for 3 h. Following this pretreatment, a 1:3 CO_2_:H_2_ gas mixture was introduced at a constant flow rate under a pressure of 10 bar. The catalytic performance was then measured within a temperature range of 245–300 °C.

**Figure 5:**
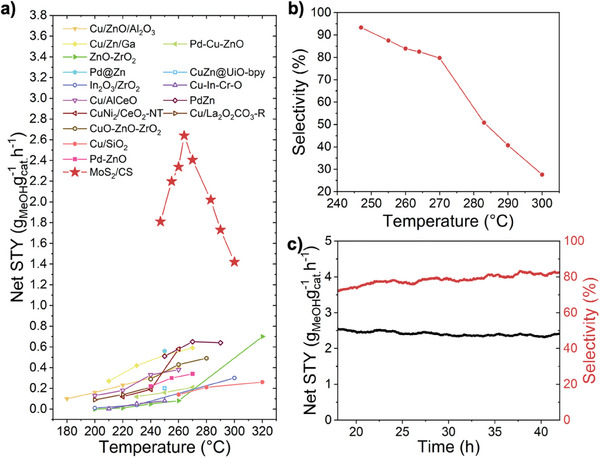
Catalytic performance of the MoS_2_/CS catalyst. a) comparison of the net space‐time yield of methanol between MoS_2_/CS catalyst and other CO_2_ hydrogenation catalysts.^[^ ^7^, ^8^, ^9^, ^10^, ^11^, ^12^, ^13^, ^14^, ^15^, ^16^, ^17^, ^35^, ^36^, ^37^, ^38^, ^39^ ^]^ b) Methanol selectivity under different temperatures. c) Stability test of MoS_2_/CS catalyst.

**Figure** **5**a presents the methanol yield measured by gas chromatography (GC) at different temperatures, peaking at 265 °C and decreasing at higher temperatures. This result is consistent with the thermodynamics of the reaction, as the hydrogenation of CO_2_ has a negative entropy change.^[^ ^40^ ^]^ According to Gibbs’ law, an increase in temperature is not favorable for a reaction with negative entropy change. Below 265 °C the yield of methanol decreases with the temperature due to the lower reaction rate. The space‐time yield of methanol was 2.68 g_MeOH_g_cat._^−1^ h^−1^, which surpasses the performance of other CO_2_ hydrogenation catalysts (Figure 5a).^[^ ^7^, ^8^, ^9^, ^10^, ^11^, ^12^, ^13^, ^14^, ^15^, ^16^, ^17^, ^35^, ^36^, ^37^, ^38^, ^39^ ^]^

In addition, the selectivity for CO_2_ hydrogenation to methanol was calculated and our catalyst achieved an exceptional methanol selectivity of 82.5% under 264 °C. The control experiment showed that the selectivity of MoS_2_/CS was particularly notable at lower temperatures (Figure 5b), even reaching above 90%. However, 264 °C was chosen as the optimal reaction temperature to balance both yield and selectivity.

Long‐term stability test demonstrated no discernible decay in activity over a 45‐h period at 265 °C (Figure 5c). The catalyst maintained a consistent methanol yield of 2.6 g_MeOH_g_cat._^−1^ h^−1^. Initially, the selectivity was ≈70%, but it increased to 82% after stabilization, indicating robust performance in both yield and selectivity over the extended testing duration. After the catalysis, EDX analysis was performed (Figure S4b, Supporting Information), where we found the Mo/S ratio preserved larger than 2, demonstrating that the sulfur vacancy was not poisoned during the reaction.

## 3 Conclusion

In this study, we successfully synthesized MoS_2_ flakes on a porous CS using a template‐assisted CVD method. The porous CS, derived from the pyrolysis of a resin‐GO core–shell network, provided a high surface area and a stable substrate for the out‐of‐plane growth of MoS_2_ flakes. Hydrogen reduction pretreatment was effectively employed to create sulfur vacancies in the MoS_2_ flakes, which are crucial active sites for the CO_2_ hydrogenation reaction.

The catalytic performance of the MoS_2_/CS composite was evaluated in a packed bed tubular reactor. The catalyst exhibited a high space‐time yield of 2.68 g_MeOH_g_cat._^−1^ h^−1^ with a selectivity of 82.5% for methanol production, surpassing the performance of many other CO_2_ hydrogenation catalysts. The optimal catalytic activity was achieved at 265 °C, consistent with the thermodynamic constraints of the reaction.

Long‐term stability tests demonstrated that the MoS_2_/CS catalyst maintained its activity and selectivity over 45 h of continuous operation, with no discernible decay in performance. The stable yield of 2.68 g_MeOH_g_cat._^−1^ h^−1^ and the selectivity increasing to 82% after stabilization underscore the robust and durable nature of the catalyst.

Overall, our results indicate that the MoS_2_/CS composite is a promising catalyst for the efficient hydrogenation of CO_2_ to methanol, offering potential for industrial applications. The combination of a high surface area carbon scaffold, controlled synthesis of MoS_2_, and strategic hydrogen reduction pretreatment contribute to the superior catalytic performance and long‐term stability observed in this study.

## 4 Experimental Section

### Carbon Scaffold Preparation

To fabricate an emulsion‐templated carbon scaffold a mixture of 250 mL of deionized water, 50 mL of aqueous GO dispersion (0.4 wt.%, Graphenea, USA), and 10 g of UV‐curable resin (polyurethane/acrylate‐based Clear Resin, Anycubic) was mixed. Lateral dimensions of graphene oxide sheets were within the 1–10 µm range. The mixture was emulsified by probe sonicator (UIP1500HDT, Hielscher) at 20 kHz, 0.33 W, for 6 min. A resin‐in‐water droplet emulsion stabilized with GO sheets was obtained. After adjusting pH to 10 by adding 0.1 m KOH, the emulsion was cured under UV light at 405 nm for 5 min under stirring which yielded a dispersion of core–shell microspheres of 5–50 µm diameter having a resin core and graphene oxide shell. The pH of the dispersion was adjusted to 5 using 0.1 m HCl to facilitate the agglomeration of GO microspheres into interconnected network. The agglomerates were collected on polyethersulfone membrane (pore size: 0.03 µm; diameter: 90 mm; Sterlitech) by vacuum‐assisted filtration. The obtained dry cake was pyrolyzed in a tube furnace (Protherm) at 700 °C (3 °C min^−1^) for 2 h under 250 sccm Ar flowrate to decompose resin cores and produce a 3D porous carbon scaffold. The core–shell microspheres were observed by SEM Supra 40 (Carl Zeiss AG, Germany). For the TEM observation (Hitachi TEM HT7830, HR mode, Acc. Volt: 100 kV), the particles were epoxy‐moulded and sliced in ultramicrotome RMC PowerTome PTPCZ (RMC Boeckeler) with the slice thickness of 70 nm.

### Growing MoS_2_ on Carbon Scaffold by CVD

CVD was done in a two‐zone tubular furnace (Protherm, USA) with 90 sccm of Ar as the carrier gas. MoO_3_ powder (99.97%, Sigma–Aldrich) and the carbon scaffold were placed in the same boat in a 750 °C zone, with MoO_3_ positioned 1 mm upstream of the scaffold. S powder (99.98%, Sigma–Aldrich) was placed separately 45 cm upstream in a 150 °C zone. Argon flow rate was 90 sccm (atmospheric pressure). Heating rates were 20 °C min^−1^ for MoO_3_ and 3 °C min^−1^ for S. Temperatures were maintained for 20 min, followed by cooling. The final product was MoS_2_/CS.

### Characterization Methods

The morphology of the MoS_2_/CS catalyst was observed by SEM (Zeiss Sigma 300) with an acceleration voltage of 5 kV. The Raman spectra were measured by Raman spectroscope (WITec Alpha 300 R). The chemical and the electronical state of MoS_2_/CS catalyst were measured by XPS (AXIS SUPRA+). The X‐ray source used in XPS analysis was Al Kα line (1486.6 eV) and the X‐ray power was set to 225.00 W and 15 kV. The survey scan was carried out under a pass energy of 160.00 eV and a step size of 1.00 eV while the narrow scan was carried out under a pass energy of 20.00 eV and a step size of 0.1. eV. The composition of MoS_2_/CS catalyst was measured by thermogravimetric analyzer (TGA, TA Instrument Discovery). The ESR spectra were measured by JEOL JES‐X320 ESR spectrometer. The specific surface area was measured in BET measurement by Autosorb iQ Station (Quantachrome Instruments) with N_2_ at 77.35 K in the relative‐pressure range P/P_0_  =  0.05–0.3. Before the BET measurement, the samples were degassing at 300 °C overnight. Conductivity of the carbon scaffold was measured by the 4‐point probe method using a pellet of 1 cm diameter and 500 µm thickness compressed with a hydraulic press at 5 ton. The instrument used in 4‐point probe method was Keithley 2401 sourcemeter. STEM/HAADF experiments were performed on a JEM ARM200F microscope with a ASCOR corrector operating at 80 kV.

The catalytic performance was evaluated by online mass spectrometry (HPR‐20 TMS, Hidden Analytical) and gas chromatography (Agilent 8890 GC). CO_2_ hydrogenation reaction was performed in a fixed‐bed tubular reactor containing 5 mg catalyst under 10 bar pressure. Before the reaction, the catalyst was reduced under 30 ml min^−1^ H_2_ at 1 bar and 300 °C for 3 h. After the reduction, the H_2_/CO_2_ mixture with a ratio of 3:1 was introduced into the reactor. The reactions were performed in a temperature range from 245 to 300 °C, with GHSV of 96000 ml g_cat_^−1^ h^−1^. The reaction products were quantified using an online gas chromatograph equipped with a thermal conductivity detector (TCD) and a flame ionization detector (FID). Hydrogen, carbon dioxide, and carbon monoxide were separated in a combination of packed HayeSep Q 80/100 column and MolSieve 5A 60/80 column and detected by TCD, while methane and methanol were separated in capillary DB‐WAX column and detected by FID. The real‐time product flow composition was additionally monitored by an online mass spectrometer to determine the stable phase of the reaction under a fixed set of experimental conditions before taking the GC measurements. Product selectivity was calculated on a molar carbon basis.

### Statistical Analysis

All statistical analyses were performed using OriginPro 2021b SR2 (OriginLab Corporation, USA). The following procedures were applied:

### Statistical Analysis—Pre‐Processing of Data

Prior to analysis, the raw data were examined for outliers using standard diagnostic plots. ESR spectra were pre‐processed using a Savitzky–Golay filter to enhance the signal‐to‐noise ratio. In cases where data distributions deviated from normality, appropriate data transformations (e.g., logarithmic transformation) were applied to meet the assumptions of the parametric tests.

### Statistical Analysis—Data Presentation

Quantitative data are expressed as mean ± standard deviation (SD). This format is used throughout the manuscript to clearly indicate the central tendency and variability of the measured parameters.

### Statistical Analysis—Sample Size

For catalytic performance evaluation and other quantitative analyses, each measurement was performed in triplicate using independently prepared samples (n = 3).

### Statistical Analysis—Software for Statistical Analysis

All statistical computations and data visualizations were carried out using OriginPro 2021b SR2 (OriginLab Corporation, USA).

## Conflict of Interest

The authors declare no conflict of interest.

## Supporting information



Supporting Information

## Data Availability

The data that support the findings of this study are available from the corresponding author upon reasonable request.
