# Supplementary material for: Enhanced CO2 Hydrogenation to Methanol Using out‐of‐Plane Grown MoS2 Flakes on Amorphous Carbon Scaffold
Source: Small. 2025 Feb 10;21(11):2408592. doi: 10.1002/smll.202408592 (PMC11922024; doi:10.1002/smll.202408592)
Supplement: Supplementary file 1 — Supporting Information [file SMLL-21-2408592-s001.docx]

Supporting Information

**Enhanced CO₂ Hydrogenation to Methanol Using Out-of-Plane Grown MoS₂ Flakes on Amorphous Carbon Scaffold**

Mo Lin, Maxim Trubyanov, Han Wei Lee, Artemii S. Ivanov, Xin Zhou, Pengxiang Zhang, Yixin Zhang, Qian Wang, Gladys Shi Xuan Tan, Kostya S. Novoselov and Daria V. Andreeva^*^

Institute for Functional Intelligent Materials, Materials Science and Engineering Department, National University of Singapore, 4 Science Drive 2, 117544, Singapore.

Email: [daria@nus.edu.sg](mailto:daria@nus.edu.sg)


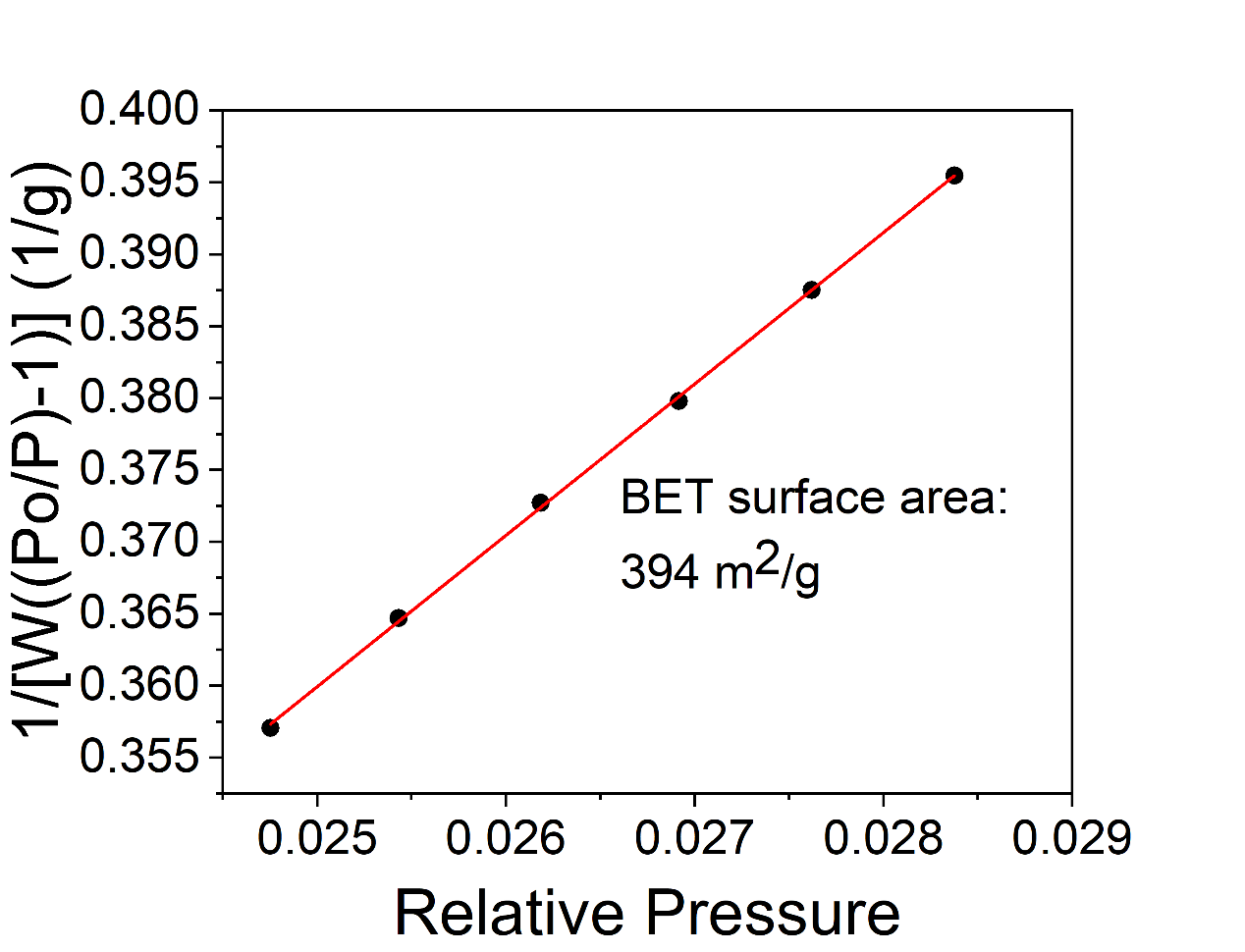


**Figure S1**. BET analysis of carbon scaffold.


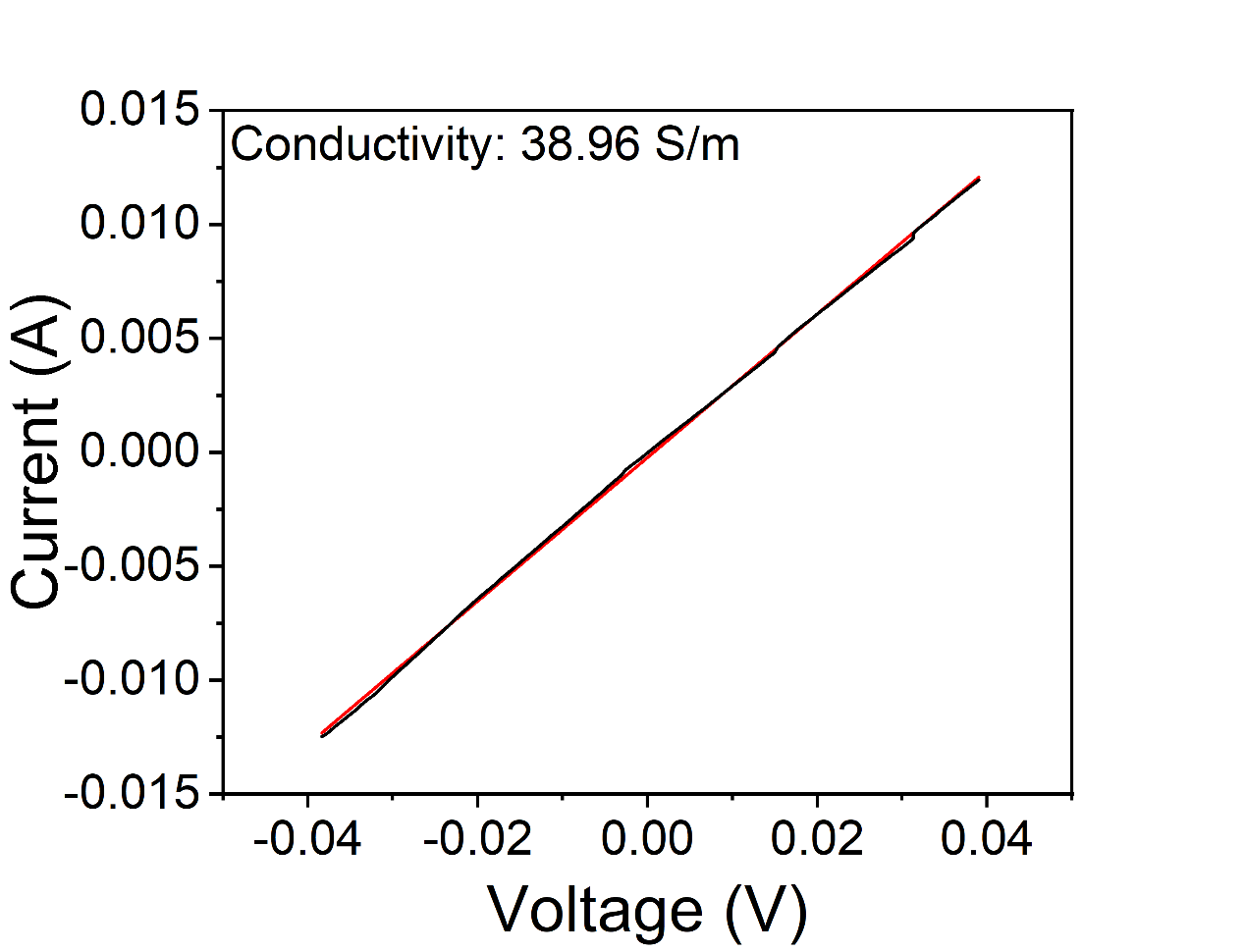


**Figure S2**. Conductivity measurement of the carbon scaffold by 4-point probe method.


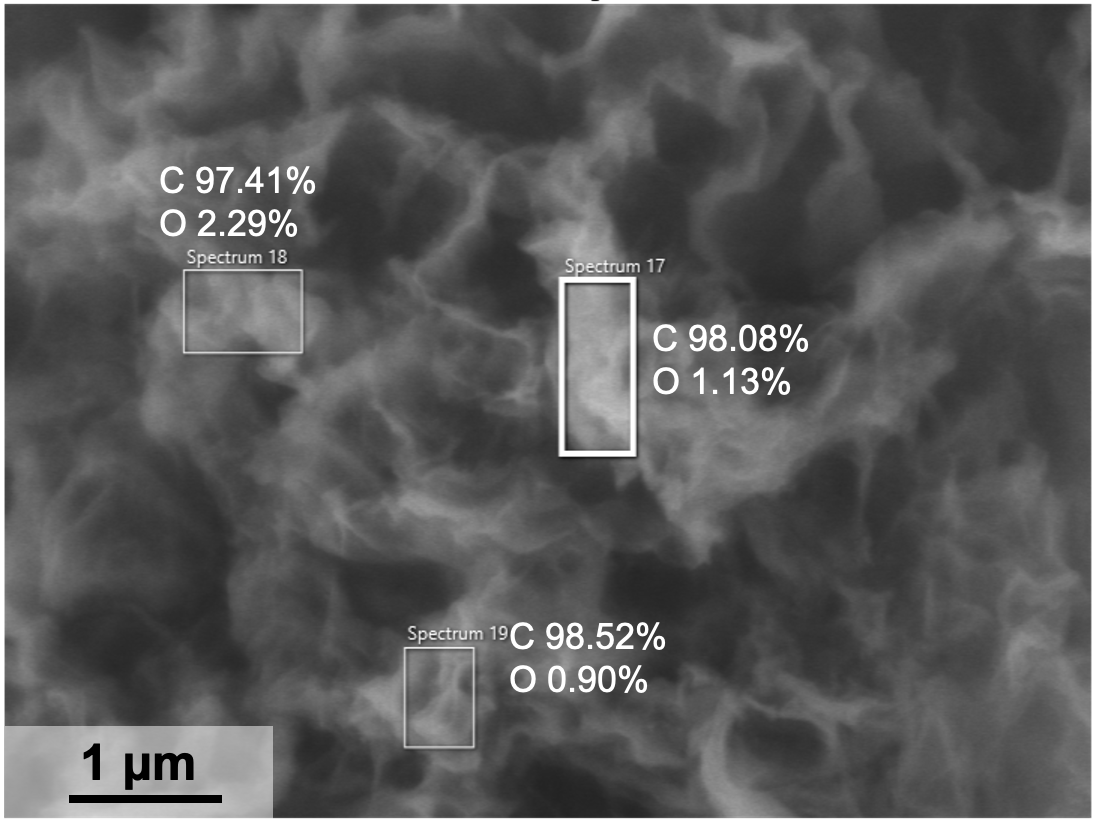


**Figure S3**. EDX data of bare carbon scaffold.


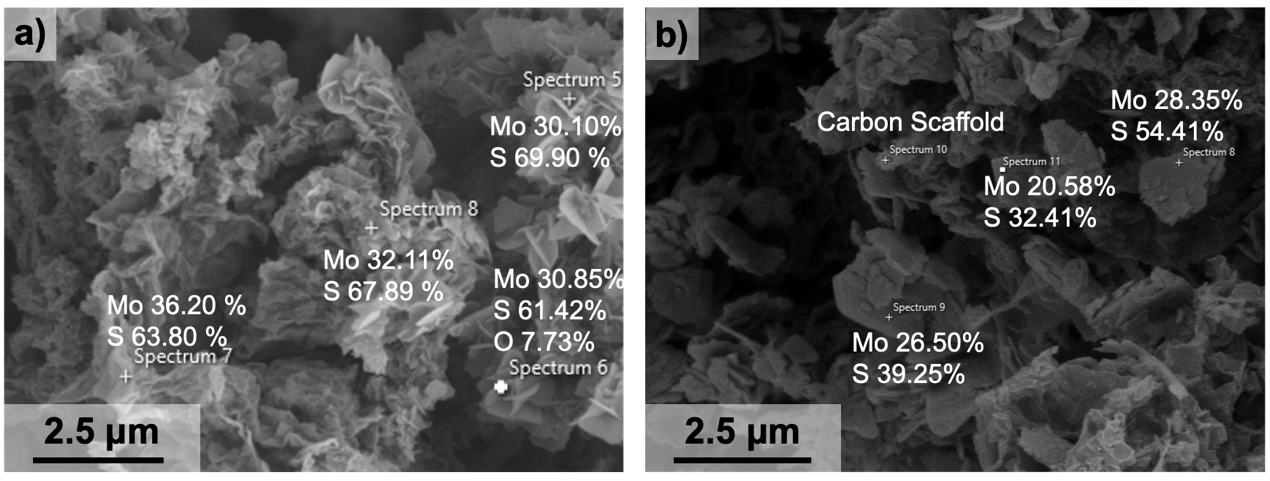


**Figure S4**. EDX data of MoS_2_/CS catalyst a) before and b) after reaction.


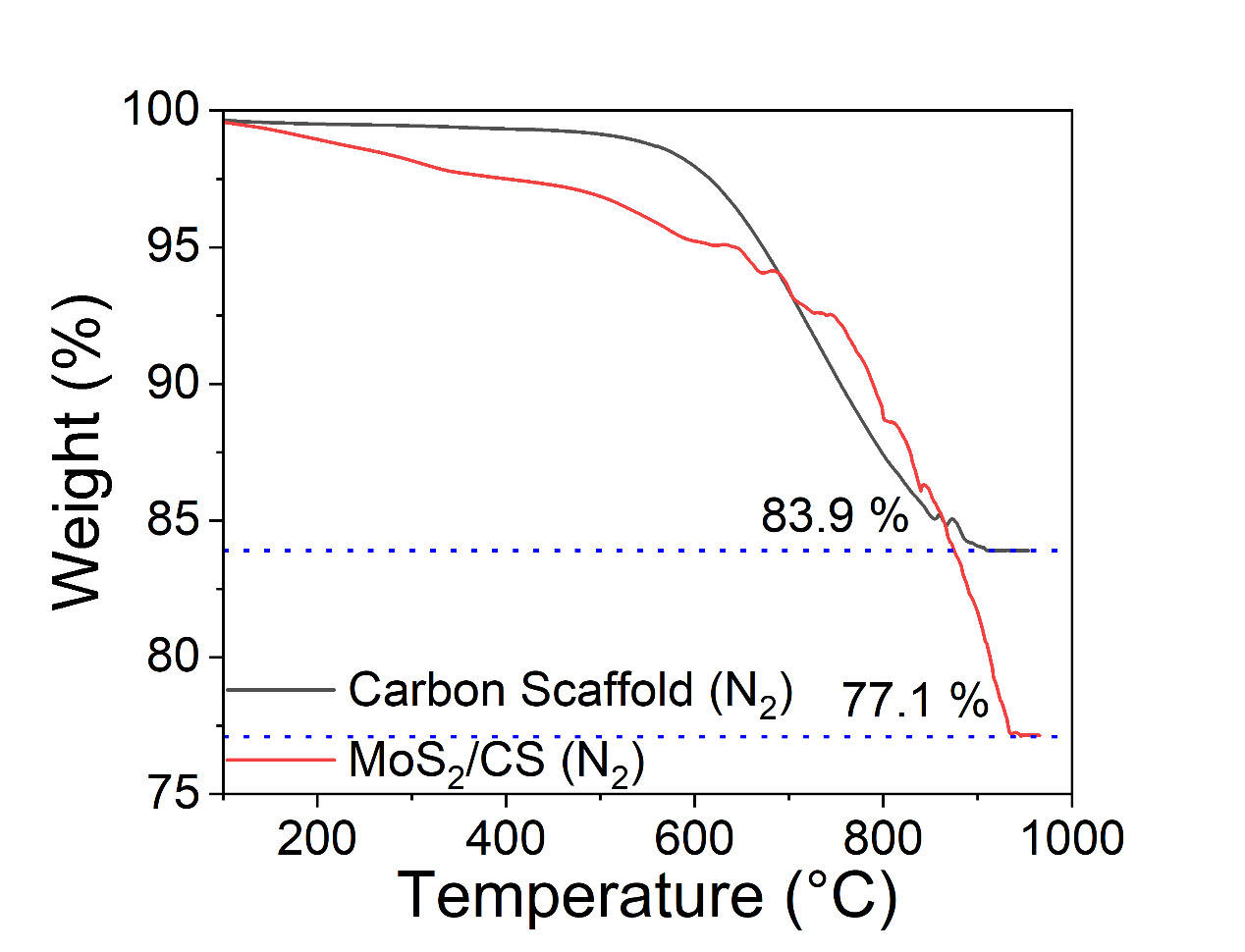


**Figure S5**. TGA curve of MoS_2_/CS and bare carbon scaffold tested under nitrogen environment.


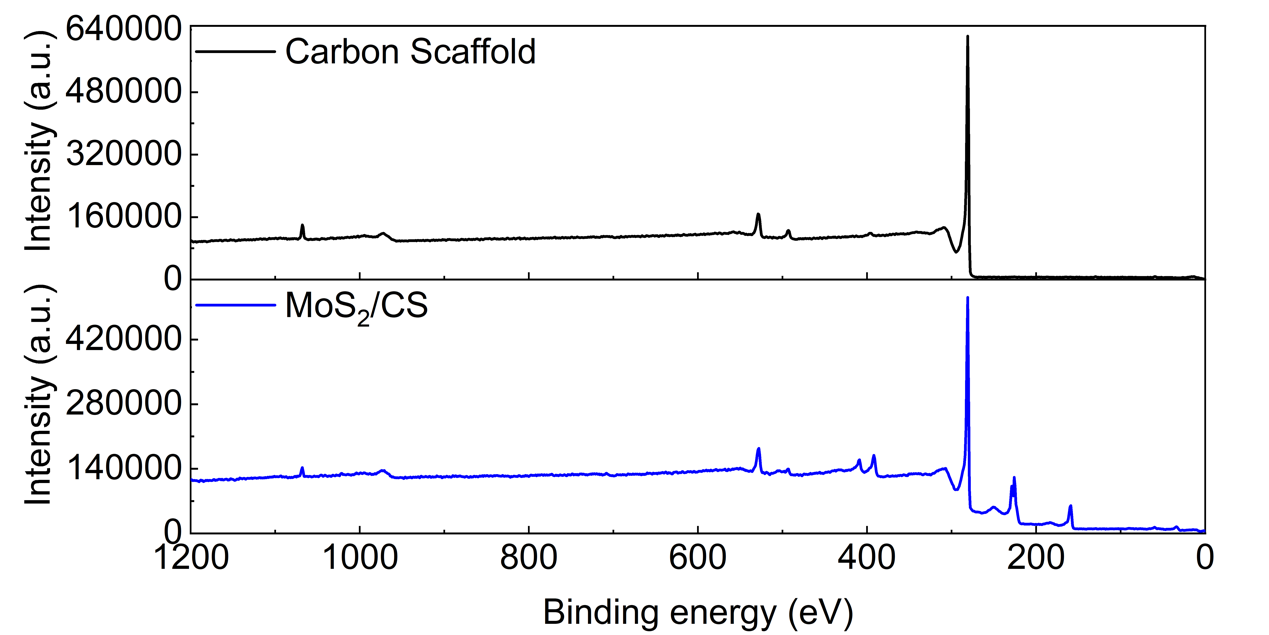


**Figure S6**. The XPS survey scan of bare carbon scaffold and MoS_2_/CS catalyst.
